# Improvements to pharmaceutical manufacturing capacity, Ethiopia 

**DOI:** 10.2471/BLT.25.295594

**Published:** 2026-09-01

**Authors:** Kedir Tahir Hagos, Tsige Gebre-Mariam, David Woo, Abraham Gebregiorgis Kahsay, Mengistab Woldearegay Teferi, Jicui Dong

**Affiliations:** a Addis Ababa, Ethiopia.; b Department of Pharmaceutics and Industrial Pharmacy, Addis Ababa University, Addis Ababa, Ethiopia.; c Product Policies, Access and Manufacturing Support, World Health Organization, Avenue Appia 20, 1211 Geneva 27, Switzerland.; d Regulatory Systems Strengthening, World Health Organization, Geneva, Switzerland.; e Essential Drugs and Medicines, World Health Organization Country Office, Addis Ababa, Ethiopia.

## Abstract

**Problem:**

Ethiopia's heavy dependence on imported pharmaceuticals created public health vulnerabilities, foreign exchange pressures and recurrent medicine stock-outs.

**Approach:**

In 2015, Ethiopia launched a 10-year National Strategy and Plan of Action for Pharmaceutical Manufacturing Development to improve access to quality-assured medicines. The strategy, overseen by a Joint Steering Committee co-chaired by the Ministries of Health and Industry with technical assistance from the World Health Organization (WHO), combined industrial incentives, preferential centralized procurement and regulatory strengthening.

**Local setting:**

Before strategy implementation, Ethiopia had limited manufacturing capacity. Local manufacturers supplied approximately 20% of national generic medicine demand, while supply chain fragmentation and foreign exchange constraints compromised access to imported medicines.

**Relevant changes:**

By 2025, six facilities had achieved national good manufacturing practice compliance, 11 manufacturers were operational and the Ethiopian Food and Drug Authority reached WHO Maturity Level 3. Locally produced coverage of the National Essential Medicines List increased from 90 to 140 chemical entities, across 167 dosage forms, and a vaccine manufacturing initiative was launched. However, domestic supply only increased to 23% against a 60% public procurement target; active pharmaceutical ingredient production and WHO prequalification remained unachieved, and export and workforce targets were missed.

**Lessons learnt:**

Combined, the strategy’s cross-sectoral governance, regulatory strengthening, preferential centralized procurement and industrial incentives advanced local production. However, foreign exchange constraints, dependence on imported raw materials and financing and capacity gaps limited progress. Future efforts should prioritize WHO prequalification, upstream manufacturing, skilled workforce development, export competitiveness and sustainable financing while maintaining patient-centred objectives.

## Résumé

**Problème:**

La forte dépendance de l’Éthiopie vis-à-vis de l’importation de produits pharmaceutiques a entraîné des vulnérabilités en matière de santé publique, des pressions sur les réserves de devises étrangères et des ruptures de stock récurrentes de médicaments.

**Approche:**

En 2015, l’Éthiopie a lancé une stratégie nationale et un plan d’action étalés sur 10 ans visant à développer sa production pharmaceutique et à améliorer l’accès à des médicaments de qualité garantie. Supervisée par un comité de pilotage conjoint coprésidé par les ministères de la Santé et de l’Industrie et bénéficiant de l’assistance technique de l’Organisation mondiale de la Santé (OMS), cette stratégie combinait des mesures d’incitation destinées au secteur industriel, un système d’achats centralisés préférentiels et un renforcement de la réglementation.

**Environnement local:**

Avant la mise en œuvre de cette stratégie, l’Éthiopie disposait d’une capacité de production limitée. Les fabricants locaux couvraient quelque 20% de la demande nationale en médicaments génériques, tandis que la fragmentation de la chaîne d’approvisionnement et les contraintes liées aux devises étrangères compromettaient l’accès aux médicaments importés.

**Changements significatifs:**

En 2025, six sites étaient conformes aux bonnes pratiques nationales de fabrication, 11 fabricants étaient opérationnels et l’Agence éthiopienne des aliments et des médicaments avait atteint le niveau de maturité 3 de l’OMS. La couverture de la liste nationale des médicaments essentiels par des produits fabriqués en Éthiopie est passée de 90 à 140 substances chimiques, réparties en 167 formes galéniques, et une initiative de fabrication de vaccins a été lancée. Toutefois, l’approvisionnement national n’a atteint que 23%, contre un objectif de 60% pour les marchés publics. La production de principes actifs pharmaceutiques et la préqualification de l’OMS sont restées inachevées, et les objectifs en matière d’exportation et de main-d’œuvre n’ont pas été atteints.

**Leçons tirées:**

Pris ensemble, la gouvernance intersectorielle, le renforcement de la réglementation, la centralisation préférentielle des achats et les incitations à destination du secteur industriel prévus par la stratégie ont favorisé la production locale. Toutefois, les contraintes liées aux devises étrangères, la dépendance à l’importation de matières premières ainsi que les déficits de financement et de capacités ont limité les avancées. Les efforts futurs devraient donner la priorité à la préqualification de l’OMS, à la fabrication en amont, au développement d’une main-d’œuvre qualifiée, à la compétitivité à l’exportation et au financement durable, tout en maintenant des objectifs centrés sur les patients.

## Resumen

**Situación:**

La fuerte dependencia de Etiopía de los productos farmacéuticos importados ha provocado vulnerabilidades en la salud pública, presiones sobre las divisas y desabastecimientos frecuentes de medicamentos.

**Enfoque:**

En el año 2015, Etiopía lanzó una estrategia nacional de 10 años y un plan de acción para el desarrollo de la fabricación de productos farmacéuticos con el fin de mejorar el acceso a medicamentos de calidad garantizada. La estrategia, supervisada por un Comité Directivo Conjunto copresidido por los Ministerios de Sanidad e Industria con el apoyo técnico de la Organización Mundial de la Salud (OMS), combinó incentivos industriales, adquisiciones centralizadas preferentes y un refuerzo normativo.

**Marco regional:**

Antes de la implementación de la estrategia, Etiopía tenía una capacidad de fabricación limitada. Los fabricantes locales suministraban aproximadamente el 20% de la demanda nacional de medicamentos genéricos, mientras que la fragmentación de la cadena de suministro y las restricciones de divisas dificultaban el acceso a los medicamentos importados.

**Cambios importantes:**

Para el año 2025, seis instalaciones etíopes habían logrado cumplir con las buenas prácticas de fabricación, 11 fabricantes estaban operativos y la Autoridad de Alimentos y Medicamentos de Etiopía había alcanzado el nivel 3 de madurez de la OMS. La cobertura de producción local de la Lista Nacional de Medicamentos Esenciales aumentó de 90 a 140 entidades químicas, en 167 formas farmacéuticas, y se puso en marcha una iniciativa de fabricación de vacunas. Sin embargo, la oferta nacional solo se incrementó hasta el 23%, frente a un objetivo de contratación pública del 60%. La producción de ingredientes farmacéuticos activos y la precalificación de la OMS siguieron sin lograrse, y se incumplieron los objetivos de exportación y de mano de obra.

**Lecciones aprendidas:**

En conjunto, la gobernanza intersectorial de la estrategia, el refuerzo normativo, la adquisición centralizada preferente y los incentivos industriales impulsaron la producción local. Sin embargo, las restricciones de divisas, la dependencia de materias primas importadas y los problemas de financiación y capacidad limitaron los avances. Los esfuerzos futuros deben priorizar la precalificación de la OMS, las fases iniciales de producción, el desarrollo de mano de obra cualificada, la competitividad de las exportaciones y la financiación sostenible, manteniendo al mismo tiempo los objetivos centrados en los pacientes.

## ملخص

**المشكلة:**

أدى اعتماد إثيوبيا الكبير على الأدوية المستوردة إلى ثغرات في الصحة العامة، وضغوط على النقد الأجنبي، ونقص متكرر في مخزون الأدوية.

**الأسلوب:**

في عام 2015، أطلقت إثيوبيا استراتيجية وخطة عمل وطنية مدتها عشر سنوات لتطوير التصنيع الدوائي بهدف تحسين الحصول على أدوية مضمونة الجودة. وقد جمعت هذه الاستراتيجية، التي أشرفت عليها لجنة توجيهية مشتركة برئاسة مشتركة من وزارتي الصحة والصناعة وبمساعدة فنية من منظمة الصحة العالمية، بين الحوافز الصناعية، والمشتريات المركزية التفضيلية، وتعزيز الأنظمة الرقابية.

**المواقع المحلية:**

قبل تنفيذ الاستراتيجية، كانت قدرة إثيوبيا التصنيعية محدودة. وكان المصنعون المحليون يغطون حوالي %20 من الطلب الوطني على الأدوية العامة، في حين أن تفتيت سلسلة التوريد والقيود على النقد الأجنبي قد أدى لإعاقة الحصول على الأدوية المستوردة.

**التغيّرات ذات الصلة:**

بحلول عام 2025، حققت ستة مرافق الامتثال لممارسات التصنيع الجيدة الوطنية، وبدأت 11 جهة تصنيع بالعمل، ووصلت هيئة الغذاء والدواء الإثيوبية إلى المستوى الثالث من معايير منظمة الصحة العالمية للنضج. وازدادت التغطية المحلية لقائمة الأدوية الأساسية الوطنية من 90 إلى 140 مادة كيميائية، عبر 167 جرعة دوائية، كما تم إطلاق مبادرة لتصنيع اللقاحات. مع ذلك، لم تتجاوز زيادة نسبة الإمداد المحلي %23 مقابل %60 من هدف المشتريات العامة؛ وظل إنتاج المكونات الدوائية الفعالة والتأهيل المسبق لمنظمة الصحة العالمية غير متحققين، ولم تُحقق أهداف التصدير والقوى العاملة.

**الدروس المستفادة:**

ساهمت الحوكمة الشاملة للقطاعات، وتعزيز الأنظمة، والمشتريات المركزية التفضيلية، والحوافز الصناعية، مجتمعةً، في تطوير الإنتاج المحلي. إلا أن القيود المفروضة على النقد الأجنبي، والاعتماد على المواد الخام المستوردة، وفجوات التمويل والقدرات، قد قللت من التقدم. ينبغي أن تُعطي الجهود المستقبلية الأولوية للتأهيل المسبق لمنظمة الصحة العالمية، والتصنيع الأولي، وتطوير القوى العاملة الماهرة، والقدرة التنافسية للتصدير، والتمويل المستدام، مع الحفاظ على الأهداف التي ترتكز على المريض.

## 摘要

**问题:**

埃塞俄比亚对进口药品的极度依赖导致公共卫生系统比较脆弱、外汇压力大且药品短缺问题层出不穷。

**方法:**

埃塞俄比亚于 2015 年推出了《国家药品生产十年发展战略和行动计划》，以提高质量有保证药品的可及性。该战略在由卫生部和工业部共同主持的联合指导委员会的监督下实施，得到了世卫组织 (WHO) 的技术支持，并整合了产业激励措施、集中采购优惠政策和监管强化措施。

**当地状况:**

在该战略实施之前，埃塞俄比亚的药品生产能力较为有限。本地制造商约可满足全国 20% 的仿制药需求，而供应链碎片化和外汇受限又损害了进口药品的可及性。

**相关变化:**

截至 2025 年，有 6 处生产设施符合国家优质生产规范，有 11 家制药企业正常运营，且埃塞俄比亚国家食品药品监督管理局获得了世卫组织成熟度 3 级认证。《国家基本药物清单》中的本地产化学实体从 90 种增至了 140 种（涵盖 167 种剂型），同时推出了疫苗生产计划。但是，国内供应比例仅增长至 23%，而公共采购目标为 60%；尚未实现活性药物成分生产和世卫组织预认证标准，且出口和人才目标亦未达成。

**经验教训:**

综合而言，该战略推出的跨部门管理、监管强化措施、集中采购优惠政策和产业激励措施等有效促进了本地药品生产。但是，外汇受限、依赖进口原材料以及融资和产能缺口仍然制约了发展。未来应在坚持以患者为中心目标的前提下优先推进世卫组织预认证、上游生产缓解、技术人才培养、出口竞争力提升和可持续融资。

## Резюме

**Проблема:**

Высокая зависимость Эфиопии от импорта фармацевтической продукции привела к уязвимости системы общественного здравоохранения, создала давление на валютный рынок и вызвала регулярный дефицит лекарственных средств.

**Подход:**

В 2015 году Эфиопия приняла 10-летнюю Национальную стратегию и план действий по развитию фармацевтического производства с целью улучшения доступа к качественным лекарственным средствам. Данная стратегия, реализацию которой контролировал Совместный руководящий комитет под сопредседательством министерств здравоохранения и промышленности при техническом содействии Всемирной организации здравоохранения (ВОЗ), сочетала в себе меры промышленного стимулирования, преференциальные централизованные закупки и укрепление регуляторной базы.

**Местные условия:**

До начала реализации стратегии Эфиопия обладала ограниченными производственными мощностями. Местные производители обеспечивали только около 20% национального спроса на генерические лекарственные препараты, а фрагментация цепочки поставок и нехватка иностранной валюты затрудняли доступ к импортным препаратам.

**Осуществленные перемены:**

К 2025 году шесть предприятий подтвердили соответствие национальным правилам надлежащей производственной практики (GMP), одиннадцать производителей начали работу, а Управление по контролю за продуктами питания и лекарствами Эфиопии достигло 3-го уровня зрелости по классификации ВОЗ. Доля препаратов местного производства, входящих в Национальный перечень основных лекарственных средств, увеличилась с 90 до 140 химических соединений в 167 лекарственных формах, и была запущена инициатива по производству вакцин. Тем не менее объемы внутреннего предложения выросли всего до 23% при целевом показателе государственных закупок в 60%, не удалось достичь целей по производству активных фармацевтических субстанций и прохождению преквалификации ВОЗ, а целевые показатели по экспорту и кадровому обеспечению остались невыполненными.

**Выводы:**

Комплексный подход, включающий межсекторальное управление, укрепление нормативно-правовой базы, преференциальные централизованные закупки и меры поддержки промышленности, позволил продвинуть местное производство. Однако нехватка иностранной валюты, зависимость от импортного сырья, а также дефицит финансирования и мощностей ограничили достигнутый прогресс. В рамках будущих усилий приоритетное внимание следует уделить прохождению преквалификации ВОЗ, развитию начальных этапов фармацевтического производства, подготовке квалифицированных кадров, повышению экспортной конкурентоспособности и устойчивому финансированию при сохранении целей, ориентированных на нужды пациентов.

## Introduction

The first pharmaceutical manufacturing facility in Ethiopia was established in 1964.^1^ Over subsequent decades the government introduced measures to regulate and stimulate the sector, most notably through Ethiopia’s 1993 National Drug Policy which sought to ensure the systematic availability, accessibility and affordability of essential, safe and efficacious medicines.^1^ Despite these efforts, the industry remained underdeveloped due to the absence of a comprehensive, long-term national vision and coordinated multisectoral planning.^1^

During the early 2010s, Ethiopia emerged as one of the world's fastest-growing economies, driven by state-led infrastructural investments under its first Growth and Transformation Plan.^2^^,^^3^ However, local pharmaceutical production was insufficient for the rising demand, while dependence on imports posed public health, national security and economic challenges. An evaluation of the industrial baseline in 2015 revealed that domestic manufacturers met just 20% of the national demand.^4^ This deficiency forced a heavy reliance on foreign suppliers for essential therapeutics, draining scarce foreign currency reserves and leaving the health-care system vulnerable to global supply chain shocks and price fluctuations. Similar dependence affected other sub-Saharan African countries. In Ghana, for example, approximately 70% of finished medicines were historically imported.^5^

In 2015, the Ethiopian government sought technical assistance from the World Health Organization (WHO) to address these vulnerabilities.^4^ This collaboration resulted in the launch of a National Strategy and Plan of Action for Pharmaceutical Manufacturing Development in Ethiopia (NSPA-Pharma 2015–2025, hereafter referred to as the national strategy).^4^ This paper reviews the progress, institutional upgrades and public health lessons emerging from its implementation.

## Local setting

Ethiopia's rapidly growing population and dual burden of communicable and noncommunicable diseases placed increasing demands on the health system.^6^ Before implementation of the 2015 strategy, medicine supply was constrained by fragmented public–private distribution networks and historically low local manufacturing capacity.^4^ Public health facilities, particularly rural health centres and primary hospitals, experienced recurrent stock-outs of essential medicines, compromising timely treatment and contributing to preventable health risks.^7^

## Approach

The national strategy covered the pharmaceutical manufacturing ecosystem, targeting local generic manufacturers, national regulatory bodies, higher education institutions and public procurement networks.^4^ The strategy integrated national public health priorities with an industrial development policy across 10 distinct strategic pillars covering quality upgrading, specialized industrial clusters, investment attraction and other measures to strengthen domestic production.^4^ A high-level joint steering committee co-chaired by the State Ministers of Industry and Health, was established to align industrial incentives, regulatory enforcements and licensing, import privileges, and public health requirements. To secure market volume and certainty of demand for domestic firms, the strategy leveraged the Ethiopian Pharmaceuticals Supply Service’s centralized procurement system, which manages over 70% of public sector medicine procurement.^4^ Financial incentives included extended corporate tax holidays, expedited customs clearance for raw materials, and a mandatory 25% price preference (under which the procurement agency tolerates up to 25% higher prices from local producers as compared to imported products) for domestic firms participating in public tenders.^1^^,^^4^

The strategy prioritized generic production to optimize national cost–effectiveness, reduce regulatory complexities, and address major therapeutic needs, backed by technical training from international partners such as WHO’s Local Production and Assistance Unit, the United States Agency for International Development and the United Nations Industrial Development Organization.^4^ By comparison, regional peers used alternative protectionist policy mixes; for instance, to protect its domestic manufacturers from low-cost Asian imports,

## Relevant changes

### Progress against targets

As summarized in Table 1, quantitative indicators show mixed progress over the 10-year implementation period. A major achievement was the establishment of the specialized Kilinto Pharmaceutical Industrial Park, providing dedicated infrastructure and services to reduce manufacturing investment and operational barriers. Currently, 11 companies manufacture finished formulations, and one manufactures empty hard gelatine capsules across 13 distinct industrial sites.^8^ A 70 million United States dollars project to establish a vaccine manufacturing plant within the park led to the establishment of ShieldVax in 2023.^13^ Five new and one existing facility achieved national good manufacturing practice compliance, raising the number of compliant sites from zero at the beginning of the national strategy to six.^9^ However, major industrial targets remained unmet. No local firm has achieved WHO prequalification, limiting participation in large, donor-funded global health tenders. Domestic active pharmaceutical ingredient manufacturing also remained entirely unfulfilled due to high capital costs, complex chemical processes and energy requirements, leaving manufacturers dependent on imported raw materials.^1^^,^^4^^,^^10^ At the same time, capacity utilization averaged only 43% across the industry, largely because foreign currency shortages constrained imports of raw materials and other inputs.^10^ Consequently, the local market share via the Ethiopian Pharmaceuticals Supply Service’s Revolving Drug Fund procurement stood at 23% in 2024, missing the 60% target.^11^

**Table 1:** Selected indicators of progress and targets of the national strategy and plan of action for pharmaceutical manufacturing development,^4^ Ethiopia

Indicator	2015 (strategy launch)	2020 mid-term target	2025 end-term target	Achieved (2025)
No. of new manufacturing companies established	NA	5	11	5^8^
No. of manufacturers with national good manufacturing practice compliance	0	5	20	6^9^
No. of manufacturers of active pharmaceutical ingredients	0	1	3	0^10^
No. of WHO prequalified products produced locally	0	4	15	0
% of essential medicines purchased by the Ethiopian Pharmaceuticals Supply Service from local firms	15–20	50	60	23^11^
Export of locally produced medicines, in US$ million^a^	<1	30	80	< 2^12^
No. of graduates in industrial pharmacy or regulatory sciences^b^	0	200	1500	~40

NA: not applicable; US$: United States dollars; WHO: World Health Organization.

^a^ Export values are based on the United Nations Comtrade Database.^12^

^b^ Data on graduates in medicines regulatory sciences were compiled from personal communications (2025, 2026) with programme coordinators at the relevant Ethiopian universities.

### Implications for patients

Expansion of local production increased the coverage of the National Essential Medicines List produced locally from 90 to 140 chemical entities across 167 dosage forms.^8^ A 30% advance payment scheme for domestic firms also helped reduce stock-out risks for commonly used medicines (including antibiotics and analgesics), particularly during periods of global supply disruption.^1^^,^^4^ Both of these developments have helped create a more consistent supply within public health facilities, which is critical for patients needing timely access to essential treatments. The growth of local manufacturing has also improved the efficiency of medicine procurement and distribution, reducing lead times in some public tenders. Importantly, improved regulatory oversight and manufacturer quality systems have reduced the circulation of substandard or falsified medicines, strengthening patient safety and treatment reliability.^14^^,^^15^ While these changes may have eased access for patients in certain contexts, broader economic pressures such as inflation and currency fluctuations continued to constrain sustained improvements in affordability.^10^ The strategy and related sectoral policies focused largely on supply and production capacity. There was less emphasis on complementary measures to promote appropriate medicine use, such as programmes to ensure responsible use of antibiotics and improve prescribing practices. Consequently, increased availability has not yet been fully matched by improvements in appropriate use at patient level.

### Benchmarking

In September 2025, the Ethiopian Food and Drug Authority achieved WHO Maturity Level 3 for the regulation of medicines and vaccines (non-producing),^14^^,^^15^ confirming that Ethiopia’s regulatory system is stable and functioning. Ethiopia became one of only nine African countries to attain Maturity Level 3 status, strengthening regulatory credibility, mutual recognition among participating authorities, reducing duplication and accelerating access to quality-assured medicines.^14^^,^^15^ Ethiopia's participation in regional economic frameworks, including the African Continental Free Trade Area and the Common Market for Eastern and Southern Africa, further enhances opportunities for regional market integration.^15^

## Lessons learnt

Several lessons were learnt from implementing the national strategy (Box 1). First, dual-chaired governance is essential for policy coherence. The joint steering committee aligned industrial policy, public health priorities, regulatory oversight and public procurement. Sustained political commitment supported cross-sectoral collaboration and implementation.

Box 1 **Summary of main lessons learnt**

1. Aligning industrial, regulatory and procurement policies under clear, cross-sectoral accountability helps convert domestic manufacturing capacity into reliable patient access.
2. Coupling international regulatory credibility (such as WHO Maturity Level 3) with secured, timely public procurement helps minimize stock-outs.
3. Ensuring access to raw materials and foreign currency for local production helps protect long-term availability and affordability of essential medicines.

WHO: World Health Organization.

Second, regulatory credibility is a powerful market enabler. Achieving WHO Maturity Level 3 confirmed that Ethiopia's medicines regulatory system was stable and well-functioning.^15^ The milestone strengthened investor confidence, enhanced the credibility of locally manufactured medicines and created opportunities for regional regulatory harmonization.^15^

Third, industrial infrastructure requires adequate market and financial support. Strategic investment in the Kilinto Pharmaceutical Industrial Park, with factory plots, infrastructure and services in place, reduced startup costs and operational barriers. Reforms enabling the centralized public procurement system to meet domestic demand further strengthened the market.^4^^,^^9^ However, persistent foreign exchange shortages constrained imports of active pharmaceutical ingredients and other inputs, limiting capacity use. Ghana similarly combined a 15% procurement price preference and negative import list with the Export Trade, Agricultural and Industrial Development Fund to support factory upgrades, but continued to face fragmented procurement and WHO prequalification barriers.^5^ These experiences demonstrate that infrastructure, market support and access to finance need to operate as complementary policy instruments.

Fourth, upstream supply chain resilience remains a strategic priority. Dependence on imported active pharmaceutical ingredients and production inputs exposes manufacturers to foreign exchange shortages and global supply disruptions. Greater upstream manufacturing, diversified raw-material sources and stronger regional supply networks are key to sector resilience and competitiveness.

Finally, the ultimate measure of success should be patient-centred, with manufacturing capacity, investment and regulatory maturity important intermediate outcomes. Ultimately, local production should improve access to affordable, quality-assured medicines and strengthen health security.

The fact that several targets remained unmet reflects implementation challenges rather than strategic shortcomings. The shortfalls should be interpreted within Ethiopia's broader macroeconomic and global operating environment; they were compounded by the coronavirus disease 2019 pandemic. Implementation was hampered by global supply chain disruptions, foreign currency shortages and the capital-intensive nature of active pharmaceutical ingredient manufacturing. In response, government institutions are strengthening investment promotion, expanding technical assistance for WHO prequalification, improving access to industrial financing, and exploring mechanisms to support upstream pharmaceutical manufacturing and greater export competitiveness. Looking ahead, Ethiopia should consolidate its gains by strengthening pharmaceutical workforce development, promoting selected upstream manufacturing and developing the next national strategy in closer alignment with universal health coverage and pandemic preparedness.
